# COVID-19 market disruptions and food security: Evidence from households in rural Liberia and Malawi

**DOI:** 10.1371/journal.pone.0271488

**Published:** 2022-08-08

**Authors:** Shilpa Aggarwal, Dahyeon Jeong, Naresh Kumar, David Sungho Park, Jonathan Robinson, Alan Spearot

**Affiliations:** 1 Indian School of Business, Hyderabad, India; 2 DIME, The World Bank, Washington, DC, United States of America; 3 University of California, Santa Cruz, CA, United States of America; 4 Abdul Latif Jameel Poverty Action Lab (J-PAL), Cambridge, MA, United States of America; 5 Center for Effective Global Action (CEGA), Berkeley, CA, United States of America; University of Naples Federico II: Universita degli Studi di Napoli Federico II, ITALY

## Abstract

We use data collected from panel phone surveys to document the changes in food security of households in rural Liberia and Malawi during the market disruptions associated with the COVID-19 lockdowns in 2020. We use two distinct empirical approaches in our analysis: (a) an event study around the date of the lockdowns (March to July 2020), and (b) a difference-in-differences analysis comparing the lockdown period in 2020 to the same months in 2021, in order to attempt to control for seasonality. In both countries, market activity was severely disrupted and we observe declines in expenditures. However, we find no evidence of declines in food security.

## Introduction

Rural Africa has largely been an afterthought during the COVID-19 pandemic, due in part to substantially lower (reported) disease prevalence. Yet while case counts have stayed relatively modest, economic disruptions were nearly as intense as those in developed countries, especially near the beginning of the pandemic [1]. What was the impact of these lockdowns on the livelihoods of rural households?

This study is set in rural Liberia and Malawi. Both countries implemented versions of lockdowns from roughly March 2020 to July 2020, though restrictions were more severe in Liberia (which ordered a full shelter-in-place for 3 months) than in Malawi (which did not impose shelter-in-place but still closed schools and placed restrictions on transportation and gatherings). Both countries restricted cross-border movement. In both countries, many services were disrupted, and we document enforcement of market closure guidelines and large declines in market activity.

The data used in this paper was collected as part of an evaluation of a large unconditional cash transfer (UCT) program that had been ongoing when the pandemic started. The project took place in 300 villages in each country, but in order to abstract away from the protective effects that were very likely imparted by the cash transfers, we restrict our analysis to the control villages, where nobody received any transfers (these make up half the study sample, i.e. 150 villages in each country). We also exclude additional 45 villages in Liberia where data collection did not overlap with COVID lockdown periods. At the beginning of the project (in 2018), 20% of the sample (2 respondents per village) were selected to take part in monthly phone surveys that began in 2019, well before the global onset of COVID-19. The survey was conducted every 2 months for each household (with half of the sample interviewed each month). The surveys continued during the lockdowns, and for more than a year after the lockdowns were first implemented. The respondents in this sample are representative of the approximately 32,000 households (150,000 people) in the study region.

The main outcome that we study in this paper is food security, which we measured both before and after the pandemic in a consistent fashion, i.e., using an identical set of survey questions and with an unchanged survey modality. We use 3 measures which are recommended for use by organizations such as the FAO and USAID, and have been validated in several settings to meaningfully correlate with food security [2]. These are (1) the household dietary diversity score (HDDS); (2) the food consumption score (FCS); and (3) the household hunger scale (HHS). In addition, the surveys included questions on income, labor supply, expenditures, transfers and other related outcomes, as well as a module on attitudes towards COVID, and resultant behavior changes, which was added in May 2020.

We use this data in two distinct empirical strategies to document how food security changed in these areas during the lockdowns. In the first, we use a similar methodology as several other studies of COVID, and use the time series from our survey data to measure the changes in food security immediately after the lockdowns were implemented relative to the period just before, i.e., an event study design.

In the second strategy, we use data collected in 2021 to implement a difference-in-differences design. Specifically, we measure changes in food security in April-August of 2020 (relative to January-February 2020) and compare them against the difference between the same months in 2021. The advantage of this second design is that it can attempt to net out seasonal differences, which are likely important in this setting in which seasonality (due especially to the harvest cycle) is an important determinant of prices and food availability. Our identifying assumption is that the effects of the lockdown on food security and prices had dissipated by 2021, such that 2021 is a valid counterfactual. We acknowledge, however, that it is possible that 2021 was also affected by COVID and other local and global macroeconomic conditions, and we view this evidence as fundamentally descriptive. Nevertheless, we argue that given the ubiquitous nature of the shock, this approach is the limit of what can be accomplished in this setting, and we argue that this topic is of sufficient importance for such evidence to be valuable.

Our main result, using either empirical design, is that we observe no evidence of a decline in food security during the lockdown. We want to emphasize that these households are food insecure to begin with: for example, the probability at baseline that at least one household member went to be hungry in the past month was 40% in Liberia and 48% in Malawi. However, these meager food security levels were preserved in the post-pandemic period. We believe that these results stem in large part from these being mostly subsistence households—while we observe a decline in food expenditures and total expenditures, we do not find a decline in food consumption itself. This is also in line with the evidence reviewed in [3], who identify two main channels through which food security was adversely impacted during the pandemic—either a reduction in income/revenues or a shortage of food in the markets. Both of these channels are largely absent for subsistence farmers.

## Methods

### Study context

This project was based on field work conducted in Liberia and Malawi between 2018 and 2021 [4]. The design was nearly identical in both countries, with minor context-specific differences. In each country, we evaluated the effect of UCTs given out by the NGO GiveDirectly (henceforth, GD). The cash transfers averaged $500 at current exchange rate (not PPP), roughly equivalent to annual household expenditures of our study households. The treatment was randomized at the village level: in treatment villages, all households received cash, while control villages received nothing. Transfers were made via mobile money; since pre-existing mobile money usage was low, beneficiaries were given the option to buy cell phones.

The study areas were chosen by implementing partners based on poverty levels, cell phone coverage, and proximity to roads. In Liberia, the project took place in 6 districts in Bong and Nimba counties; in Malawi, it took place in Chiradzulu and Machinga districts in the Southern Region. In Liberia, the project was phased in over 2 years, and we utilize only the second wave for this project (because data collection for the first wave does not coincide with the lockdown period). This wave, covering 210 villages in Bong and Nimba counties, began enrolling in early 2020. However, due to COVID-related disruptions, many villages were not enrolled until late August. In Malawi, all villages were enrolled in 2019. S1 Fig shows a timeline of project activities.

We drew a sample using information provided by GD. To select villages, GD visited each village considered for study inclusion, where GD field staff marked each habitation structure with a GPS pin. We randomly selected 10 households from this list of GPS pins, and we selected the female head of household for surveys, i.e., the spouse or partner of the male head of household was interviewed. The fact that a preponderance of our respondents is made up of women is potentially advantageous in this context as the main outcome of interest is household food consumption, a subject about which women are likely to have the most accurate information [5].

In total, 600 villages were sampled (300 in each country—the relevant second wave in Liberia included 210 of these 300), and we attempted to enroll 10 households per village in the data collection study. For this particular paper, the relevant sample is made up of 255 villages (150 villages in Malawi and 105 villages in Liberia) as we exclude villages who received cash transfers to be able to document the effects of the lockdowns for the *average* household in these areas.

As described below, our main outcome is food security. In both countries, food security follows a seasonal pattern. S2 Fig shows the harvest cycle in both countries. In Malawi, planting occurs in October-November, and the harvest occurs around May-June; in Liberia, planting starts around April and the harvest starts around September. Prices follow this same pattern, especially in Malawi. S3 Fig uses data from the World Food Programme to show that prices tend to rise in Liberia during the April-August period, while in Malawi prices tend to fall due to the harvest.

### Household phone surveys

Our evaluation was designed to measure the time-varying effects of cash transfers. We randomly selected 2 households per village to receive cell phones (worth $10–15), and enumerators called them every 2 months for approximately 2 years. The sample was drawn such that 1 household per village was called in even-numbered months, and the other in odd-numbered months (so we have 1 household per village per month, and a panel for every household at 2-month frequency). These surveys were wrapped up in August 2021 for Malawi and October 2021 for Liberia.

In addition to food security, the phone survey also included questions on income, expenditures, transfers, savings, and related outcomes. We have 3 measures of food security: (1) the household dietary diversity score (HDDS), for which foods are grouped into 12 categories, and the enumerator queries the respondent about each individual category, recording whether at least one food item in each category was consumed in the past 24 hours, summarized into an index ranging from 0–12; (2) the food consumption score (FCS), which is similar to HDDS but measures frequency of consumption rather than just indicators for 9 food groups (over the past 7 days), and ranges from 0–112; and (3) the household hunger scale (HHS), which is based on a series of 6 questions such as “In the past 4 weeks (30 days), was there ever no food to eat of any kind in your house because of lack of resources to get food?” and “In the past 4 weeks (30 days), did you or any household member go to sleep at night hungry because there was not enough food?” and ranges from 0–6.

Shortly after the lockdowns began, we expanded the scope of phone surveys to include modules geared towards measuring the impact of the unfolding crisis. These surveys started in May 2020 after IRB approvals. First, we asked a series of questions about knowledge, attitudes and behavior changes around COVID. Second, we added modules to retrospectively measure outcomes that had not been measured previously. Specifically, we added questions on spousal labor income as well as business outcomes. To construct a comparison month, we measured these month-by-month from February 2020 to May 2020 in the May/June 2020 round of the surveys (recall that half the phone survey sample was interviewed every other month) and for the month preceding the survey during survey rounds thereafter. While we administered the redesigned surveys to everyone in the phone survey sample, this paper reports results only for the GD control group in order to be able to abstract away from any protective benefits afforded by the cash transfers, which would make the results less generalizable.

S1 Table shows attrition from the household phone surveys. Completion rates ranged from 49%-82% in Liberia and were higher, 77%-100%, in Malawi. This is likely because the cellular network is stronger in rural Malawi. In general, completion rates trended downwards over time, as respondents changed phone numbers or decided to opt out of the surveys. There is no break in completion around the COVID lockdown period (March—July 2020), so sample composition changes around the lockdown are unlikely to drive our results.

Nevertheless, attrition does affect the external validity of our results. In both countries, some households never participated in the phone surveys. In Liberia, of the 206 control households, only 150 ever participated in the phone surveys; in Malawi, of the 297 control households, 285 ever participated. It is these 150 households in Liberia and the 285 in Malawi that form our analysis sample for our first empirical specification—the event study design. In S2 Table we show regressions where the dependent variable is an indicator for appearing in the event study analysis sample. We regress this indicator in bivariate regressions on 16 household characteristics as shown in the table. In Liberia, 2 characteristics are significant at 5% (access to a mobile phone, and the net value of financial assets), and 3 more at 10% (age and 2 indicators for food insecurity). In Malawi, 3 are significant at 5% (gender, an indicator for having a thatch roof and the value of land and housing), and 1 is significant at 10% (monthly expenditure). We conclude that we therefore see some deviation from the representative sample we constructed at baseline. In particular, those in the sample are somewhat better off, especially in Malawi (although the effect of land and housing assets, while significant, is minuscule), and more likely to own a phone in Liberia (where the network is poor—recall that we gave every respondent a cell phone, so this effect is not mechanical).

Our second empirical design utilizes difference-in-differences regressions in which we include only those households who are present in the data for that calendar month in both 2020 and 2021. We show correlates of inclusion into this analysis in S3 Table. In this table, the dependent variable is the percentage of survey rounds in which the household participated (which requires having data for that month in both 2020 and 2021). In Liberia, we see 3 coefficients significant at 5%: gender, household monthly expenditure, and access to a cell phone. In Malawi, we also see 3 coefficients significant at 5%: monthly food expenditure, home ownership, and value of physical assets. The size of these coefficients is modest, however: for example, in Malawi a 1 standard deviation increase in food expenditure is correlated with an increase in the proportion of completed survey rounds by 2 percentage points on a base of 85 percent.

While the pattern of coefficients in S2 and S3 Tables suggests non-random attrition, it is not clear in which direction this would bias results, and we find no qualitative difference in results between the event study and difference-in-differences specifications, suggesting that attrition is not the driving force between our findings (see below). That said, it is possible that results would differ slightly in a fully representative sample.

### Phone surveys with food vendors

In parallel to the household phone surveys, we also collected data on prices in markets near the cash transfer evaluation, and in comparison markets. There were a total of 80 markets in Liberia and 95 in Malawi. We enrolled a set of vendors in each market in a price data-collection exercise in which vendors were called once a month. In Liberia, the items were salt, imported rice, cassava, cassava flour, chicken, fresh fish, dried fish, palm oil, okra, and onions. In Malawi, the items were salt, sugar, sweet potatoes, rice, maize, maize flour, chicken, dried fish, beans, groundnuts, tomatoes, eggs, and onions. We use this data to construct market prices in order to show the impact of the pandemic on food prices as this could be a key channel through which food security would be impacted.

### Ethical considerations

This protocol was approved by the IRBs of the University of California, Santa Cruz, the University of Liberia, and the Malawi National Committee on Research in the Social Sciences and Humanities (NCRSH). We obtained written informed consent (in-person) from all respondents in the study.

### Inclusivity in global research

Additional information regarding the ethical, cultural, and scientific considerations specific to inclusivity in global research is included in the S1 File.

## Results

### Summary statistics

Table 1 presents summary statistics from our baseline survey. Because we want this paper to be able to speak to the average lockdown experience, we focus our analysis only on the cash transfer control group and present statistics for that group only.

**Table 1:** Household summary statistics.

	Liberia		Malawi	
	Mean	SD	Mean	SD
**Panel A: Demographics**				
=1 if female	0.75		0.95	
Age	43.09	14.79	38.76	14.09
=1 if currently married or has partner	0.82		0.67	
Years of education	3.28	3.86	4.95	3.42
Number of household members	4.66	2.35	4.77	2.15
**Panel B: Expenditure and assets**				
Household monthly expenditure	53.94	45.67	44.57	55.89
Household food expenditure	22.25	16.25	14.93	15.18
=1 if respondent has access to mobile phone	0.31		0.29	
=1 if house owned	0.63		0.86	
=1 if house has thatch roof	0.16		0.50	
Total value of land and housing	218.61	334.27	1,353.91	2,068.89
Total value of physical assets	10.03	30.12	85.29	118.36
Net value of financial assets	3.43	20.08	-3.74	14.56
**Panel C: Food security**				
*For any household member in the past month:*				
=1 if skipped a meal	0.37		0.38	
=1 if went to sleep hungry	0.32		0.46	
=1 if had no food for an entire day	0.16		0.28	
Observations	150		285	

Notes: Data comes from baseline surveys conducted in November—December 2019 in Liberia, and April—July 2018 for Malawi. Sample includes GiveDirectly control households only. All monetary values are in USD and winsorized at the 99th percentile. Exchange rates used for calculation are 733 Malawian Kwacha (MWK) = 1 USD and 198 Liberian Dollars (LRD) = 1 USD (May 14, 2020).

From Panel A, the vast majority of the sample was female (since we purposefully selected female heads of household for the interviews), and the average respondent was 39–43 years old. Most respondents were partnered and the average household had 4–5 members. Please note that while we interviewed female heads of households, our sample was not restricted to *female-headed* households—in Liberia, 82% of the respondents reported having a partner at the time of the interview, and in Malawi nearly 70% reported having one. Panel B shows data on expenditures, and assets. The average household spent about US $45–54 per month in expenditures, i.e., less than $0.40 a day per capita.

The average household had about $220 in assets in Liberia and $1,400 in Malawi, but the majority of this was in the form of land and housing. Other non-land assets (durable goods, livestock and financial assets) were only $10 in Liberia, and $85 in Malawi. Financial assets were almost non-existent: household net financial wealth was negative in Malawi and only $3.43 in Liberia.

Panel C documents food security. While our main results will show indices as described above, we present some intuitive components of those indices here, since they are more understandable. We find that 37% of respondents reported that someone in their household had skipped a meal in the past month because there had not been enough food, and 16–28% experienced no food for an entire day.

In order to contextualize our study sample, in S4 Table, we use data from publicly available large-scale household surveys for both countries—the 2016 wave of the Household Income and Expenditure Survey (HIES) for Liberia and the 2019–2020 wave of the Fifth Integrated Household Survey (IHS5) for Malawi—to show that while our sample is selected in very specific ways (as described in the “Study Context” section) and is not meant to be fully representative, these households are still fairly representative of not just the study areas, but also of the rural populations in their respective countries, in general.

### Documenting market disruptions

Liberia’s response to COVID was typical for Africa. Following the first case on March 16, 2020, the country immediately banned entry from countries with more than 200 cases, closed schools, and restricted public transportation. On March 21, the government announced a state of national health emergency, placing restrictions on all gathering places, including markets. On March 24, Montserrado and Margibi counties (including the capital) were ordered to shelter in place. Overland borders were closed. On April 8, the shelter-in-place was extended to the counties of Nimba, and Grand Kru, and to the entire country on April 24. Restrictions were removed on July 22.

Malawi’s response was more atypical, due to a legal challenge upheld by the country’s High Court. The government announced a “state of disaster” on March 20, 2020, which mandated school closures, restrictions on public gatherings and on travel. On April 1, the border with Mozambique closed. On April 14, the government announced a country-wide lockdown (due to start on April 18), but this order was challenged and was overturned by the High Court on April 19. Without a country-wide lockdown, Malawi’s response was one of the weakest in Africa, scoring 57/100 (compared to 88/100 for Liberia) on the Oxford COVID-19 Government Response Tracker [1]. S4 Fig displays the timeline for each country government’s responses to the onset of the pandemic in March-July 2020.

As of this writing (April 2022), Liberia has reported about 7,500 cases and 300 deaths (population of about 5 million), while the corresponding numbers in Malawi are 85,000 and 2,600 (with a population of about 18 million). New cases and deaths have largely petered out by this time.

Table 2 documents disruptions in overall economic activity after lockdown measures were implemented by the national governments during March-July 2020. Panel A shows that in Liberia, all activities were almost universally restricted. The extent of disruptions was much smaller in Malawi, but nevertheless schools, religious centers and public transportation were restricted or closed.

**Table 2:** Disruptions.

	Liberia		Malawi	
	Mean	SD	Mean	SD
**Panel A: Economics activities**				
*=1 if following places/activities were closed/restricted*:				
schools (e.g. public, private, universities, colleges, etc.)	0.99		0.99	
markets	0.96		0.16	
retail shops	0.94		0.12	
restaurants	0.97		0.20	
entertainment centers (e.g. bars, clubs, betting centers, etc.)	0.98		0.28	
religious centers (e.g. churches and mosques)	0.89		0.74	
barber shops, beauty salons	0.96		0.12	
supermarkets	0.96		0.17	
gas stations	0.93		0.10	
public transportation	0.94		0.67	
street selling	0.93		0.18	
mobile money agents	0.92		0.12	
**Panel B: Behavior changes**				
*=1 if*:				
traveled less to shops or markets	0.94		0.53	
started wearing a mask	0.84		0.32	
stopped shaking hands	0.98		0.95	
washed hands more often	0.96		0.95	
cleaned things I touch more often	0.75		0.53	
stopped going to religious services	0.91		0.58	
kept social distance from people	0.97		0.85	
Observations	779		1274	
**Panel C: Business disruptions on crop vendors**				
*=1 if*:				
closed or reduced business hours	0.98		0.25	
inventory spoiled	0.23		0.18	
consumed inventory for myself	0.44		0.12	
supply source changed	0.33		0.09	
Change in supply price from Feb to Now (%)^a^	38.25	40.27	22.57	47.19
Observations	654		1021	

Note: Means reported and standard deviations in parentheses. Data comes from first survey after COVID disruptions (in May-July 2020). Panel A and B sample includes both food vendors and households, while Panel C includes food vendors only.

^a^ This is calculated from the reported cost of procuring a fixed bundle of items February 2020 versus when the survey was conducted, which ranges from May-July 2020.

Panel B summarizes self-reported behavior changes around the start of the pandemic. Almost everyone in both countries reported that they stopped shaking hands, started washing hands more frequently, and followed social distancing norms. A significant fraction of people reported limiting travel and wearing masks. S5 Table shows a few other selected indicators. Respondents were universally aware of the virus, and levels of concern about it were quite high (this is true even in Malawi where public health measures were more muted). Respondents overwhelmingly trusted information coming from the government, and took the virus as a serious threat. However, from Panel C, no households in Malawi reported any assistance to cope with the crisis (we did not collect this data for Liberia).

Panel C presents economic disruptions as reported by food vendors. Again, the disruption was felt more strongly in Liberia, where 98 percent of vendors reporting that they were closed or reduced business hours, relative to 25 percent in Malawi. Vendors reported difficulty sourcing supplies, and reported that the cost of stocking the same bundle of supplies as they had done in February would cost 38% more in Liberia and 23% more in Malawi. S6 Table shows statistics on income losses, using retrospective data. We find large reductions in profits in Liberia, declining more than 50% in April relative to the February levels, and smaller but still substantial losses in Malawi of about 40% in April and 20% in June.

### Food security

**Fig 1:**
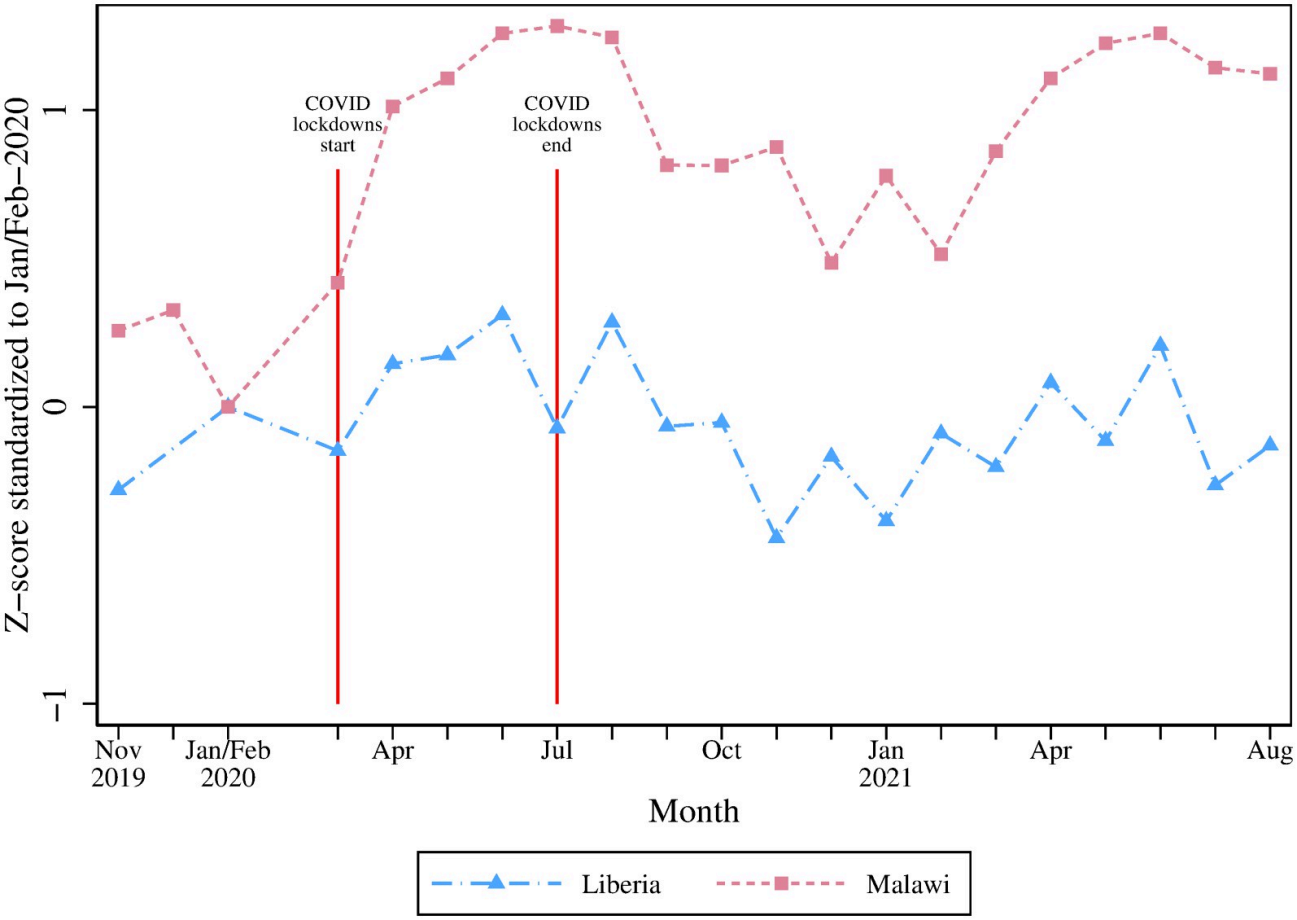
Trends in household food security index (z-score). Note: Food Security Index is a standardized z-score of HDDS, FCS, and HHS (negatively weighted), using inverse covariance weighting [6] with the mean and standard deviation for January/February 2020.

We start by presenting a time series of the food security index for both countries, starting well before the beginning of the lockdowns (November 2019) and continuing until the end of our data collection in August 2021. This series is plotted in Fig 1 and provides visual evidence that neither country experienced a worsening of food security during or after the lockdowns. In a simple pre-post comparison, food security actually improved in Malawi and was mostly unchanged in Liberia.

To analyze this data rigorously, we utilize our phone survey data in two distinct empirical approaches: (1) an event study around the date of the lockdown in March 2020; and (2) a difference-in-differences analysis comparing the same calendar months in 2020 and 2021. For the event study, we restrict analysis to data collected between January and August 2020 (except March 2020, which was only partially affected). We run the following specification using January-February 2020 as a reference group:

$$\begin{array}{c} y_{imt}=\sum_{m=Apr}^{Aug}\beta_{mt}D_{mt}+\mu_{i}+\varepsilon_{imt}, \end{array}$$ (1)

where *D*_*mt*_ is a dummy variable for month *m* in year *t* and *μ*_*i*_ is a household fixed effect. The outcome variable *y*_*imt*_ is a composite food security index (“FSI”) of household *i* in month *m* in year *t* of three different indices of food security—HDDS, FCS, and HHS—and higher values indicate greater food security. Please note that since lower values of HHS means higher food security, the FSI includes the inverted value of the HHS. *β*_*mt*_ represents the difference in food security between pre- and post-COVID lockdowns.

For the difference-in-differences specification, we add data from those same calendar months in 2021 (January-August, but again excluding March), and run the following, with standard errors clustered at the village level:

$$\begin{array}{c} y_{imt}=\alpha D_{2020}+\sum_{m=Apr}^{Aug}\left(\gamma_{m}+\beta_{m}D_{2020}\right)D_{m}+\mu_{i}+\varepsilon_{imt}, \end{array}$$ (2)

where *D*_2020_ is a dummy variable, taking a value of 1 for year 2020 and 0 for year 2021. *γ*_*m*_ captures seasonality for a particular month as an average across the two years. The coefficient on the interaction term, *β*_*m*_, is a vector of difference-in-differences estimators that compare the change in food security between January/February and month *m* in 2020 to the change over the same period in 2021. The idea is to use the year 2021, when there were no market disruptions due to COVID restrictions, as a comparison group of the year 2020. We acknowledge, however, that it is possible that 2021 was also affected by COVID and other local and global macroeconomic conditions, and may therefore have limited validity as a counterfactual. The evidence from the difference-in-differences specifications should therefore be viewed in conjunction with the event study to get a fuller picture of food security during this period.

Finally, in order to account for endogenous attrition, we run a third specification, similar to Eq 2, but with the addition of a household-calendar month fixed effect. Doing so ensures that the estimated coefficient for each month is based on the support of only that sample which is present in the data in that calendar month for both the years—2020 as well as 2021. Therefore, the regression we estimate is the following:

$$\begin{array}{c} y_{imt}=\alpha D_{2020}+\sum_{m=Apr}^{Aug}\left(\gamma_{m}+\beta_{m}D_{2020}\right)D_{m}+\mu_{im}+\varepsilon_{imt}, \end{array}$$ (3)

**Fig 2:**
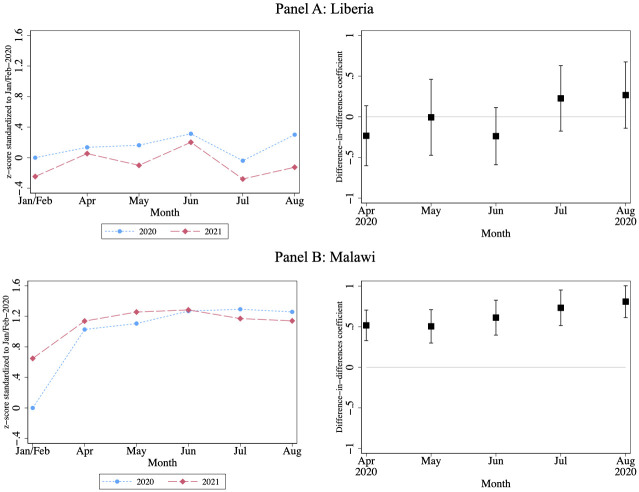
Household food security index (z-score). Note: Food Security Index is a standardized z-score of HDDS, FCS, and HHS (negatively weighted), using inverse covariance weighting [6] with the mean and standard deviation for January/February 2020. Data collected in March are excluded because March 2020 was the start of the pandemic. The regression results for this figure are reported in S7 Table. Subfigures on the left plot the change in levels across months, while those on the right report coefficients (with 95% confidence intervals) from the difference-in-differences specification in Eq 3. Regressions include household-by-calendar fixed effects. Standard errors are clustered at the village level.

We present regression results separately for Liberia and Malawi graphically in Fig 2. For each country, the left-hand panel shows the event study estimates for each year and the right-hand panel shows the difference-in-differences estimates. The difference-in-differences estimates are based on the specification given in Eq 3.

Food security in 2020 is denoted in the blue dotted lines, and shows that food security actually improved during COVID lockdown months (i.e. months starting from April 2020) relative to the pre-COVID period (i.e. January and February 2020). The increase is 0.10–0.39 standard deviations (excluding July) in Liberia, while it is 1.0–1.4 standard deviations in Malawi. However, much of this change is due to seasonality, as can be seen in the red dashed line, which denotes food security in 2021. The 2021 figures show an increase over the same time period, showing that that the jump between Jan/Feb and the subsequent months we see in 2020 can partially be explained by seasonality.

To difference this seasonal trend out, we implement the difference-in-differences in which we compare changes in 2020 to 2021. The difference-in-differences estimates on the right show that controlling for trends mitigates observed changes, but does not turn estimates negative. In fact, if anything, food security improved significantly in Malawi (0.5–0.8 standard deviations).

We present corresponding regression results for both the event study and the diff-in-diff design in S7 Table. Panel A shows Liberia and Panel B shows Malawi. Columns 1 and 4 show the event study for each country, and are followed by difference-in-differences specifications with and without household-month fixed effects. From the event study, we see an increase in food security of 0.10–0.39 standard deviations during the COVID lock-down period (except for July 2020) in Liberia, and an even bigger rise of more than 1 standard deviation in Malawi. From the difference-in-differences estimates, we see that this increase is partially seasonal—effects are attenuated in both countries—in Liberia, all months are indistinguishable from zero, and some coefficients are even negative. Similarly, the estimates are smaller in Malawi but still substantial. This latter result could be attributed to a stronger harvest in Malawi in 2020—the maize harvest in 2020 was 11% larger than that in 2019, and 28% larger than the 5-year average for the country [7].

Finally, we present results for each component of the Food Security Index separately in S5–S7 Figs. This is noteworthy because two measures (HDDS and FCS) primarily measure dietary diversity, while the third (HHS) is a measure of the quantity of food consumed. However, we find similar results for each of these three measures.

We complement our main food security outcome, FSI, with food and household expenditure in S8 and S9 Figs. While the *levels* of food expenditure did not decline in either country, we do see some evidence of declines when accounting for seasonality in the difference-in-differences (especially in Liberia). In Liberia, food expenditure declines by 5–10 USD per month until August. In Malawi, point estimates are negative but not significant, and average less than 5 USD. Total expenditures, by contrast, in S9 Fig, did not decline in either country. One possible explanation for the difference between the expenditure and food security results is that households switched to lower quality foods, or to consumption from home production. While we cannot definitively determine the mechanism here, this result does speak to how food expenditures may not always be a good proxy for food consumption.

### Prices

**Fig 3:**
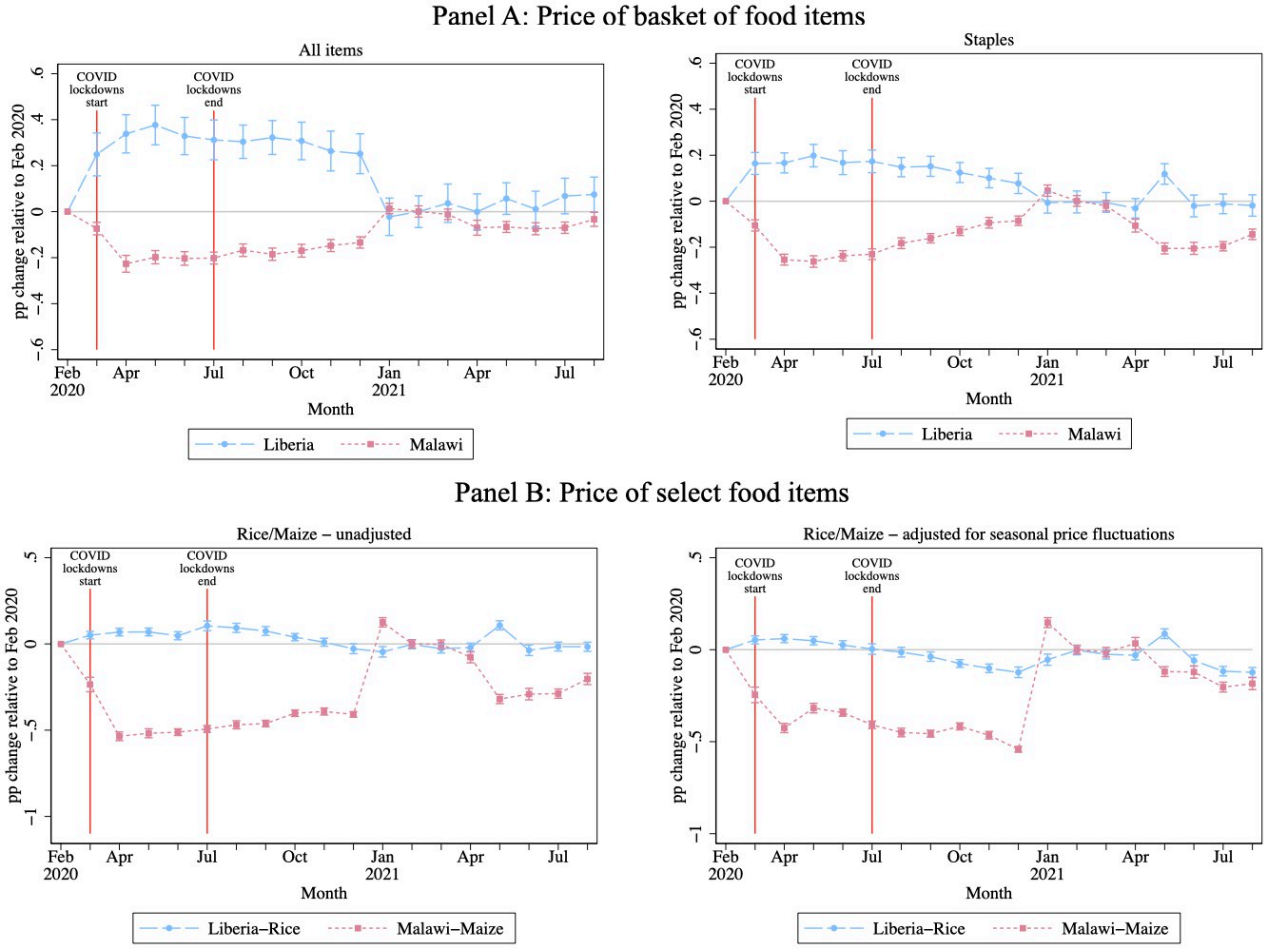
Effect on crop prices. Note: The unit of observation is the market-month. There are 95 markets and 13 products in Malawi and 85 markets and 10 products in Liberia. The figure presents coefficients from a regression on ratio of price to price in February of same year, omitting the reference period of February 2020. All monetary values are in USD and Winsorized at 1% and 99%. Standard errors are clustered at the market level. See text for crops included in analysis. Panel B shows (imported) rice and maize prices, with and without subtracting off monthly average relative prices from the WFP data (only available for these two products). All specifications include market fixed effects ans standard errors clustered at market level. Regressions for staples additionally include product fixed effects.

Fig 3 shows effects on food prices (the data series in blue is for Liberia and that in red for Malawi). This figure show deviations in prices from the reference period of February 2020.

Each panel shows different products or baskets of products. The top row of the Figure shows (1) the expenditure share weighted price index (weighted from the baseline survey) of all items (top left); and (2) staples only (top right). The bottom row makes use of historical time series data on prices taken from the WFP to subtract off monthly average prices. However, this is only possible for the staple foods of rice (Liberia) and maize (Malawi), and so in the panel we show results with and without subtracting off this average. In each plot, the figure shows point estimates and confidence intervals relative to February 2020.

The figure shows that prices increased modestly in Liberia and actually declined in Malawi. This latter effect is due in part to the seasonal maize harvest, which occurs around this time (as can be seen in the 2021 data). We show these results in formal regressions in S8 Table. In a difference-in-differences specification comparing 2020 and 2021, we observe price increases of 9–19% in Liberia for all crops (somewhat higher for staples, though lower for rice alone), and modest declines in Malawi.

### Income

We examine how income was affected in S9 Table. The odd-numbered Columns show the event study and the even-numbered Columns show the difference-in-differences. The tables show business profits (Columns 1–2), labor income (Columns 3–4), and total non-agricultural income (Columns 5–6). Our data shows that people report earning very little income, and so there is little to lose—the bottom of the Table shows that monthly mean pre-lockdown income was only about $7 in Liberia and $8 in Malawi. While it is possible that income is not comprehensively measured (especially spousal income), we nevertheless do not see any evidence of large declines. It is also worth noting that small declines in income—even for these poor households—may not necessarily translate into consumption declines for bare necessities such as food, as households will likely reallocate their budget away from discretionary expenditures to preserve food consumption.

## Relationship to existing literature and discussion

Because countries like Malawi and Liberia are so poor and lack formal safety nets, many commentators initially expected devastating effects of the lockdowns, warning of millions being pushed into poverty and even of imminent starvation [8–10]. Given the contrast with popular discourse, the preservation of food security levels during the pandemic was not expected by the research team. However, emerging evidence from academic studies suggests that perhaps the worst fears from the initial days of the pandemic were unfounded—while many studies show significant declines in food security in the aftermath of the lockdowns, there are many others who find more modest effects. In this section, we review some of these findings and discuss how our paper relates to the broader literature.

A number of prominent papers on this topic find negative effects of the pandemic on food security. [11] construct a sample of 16 surveys in 9 countries in Africa, Asia, and Latin America, and find substantial declines in employment, income, and food security in all settings; [12] find evidence of a decline in food security using panel data in Nigeria, and [13] provide similar evidence for Mali. A number of other studies also show declines in food security in many developing countries (e.g. [14–19]). [20] provide a review of the evidence generated in the immediate aftermath of the pandemic).

Why do our results differ from these studies? It is of course entirely possible that the context of Liberia and Malawi is simply different. However, we conjecture that another possibility is that ours is one of the few studies to consistently measure food security before and after the crisis, using internationally accredited measures, rather than retrospective questions about food security after the lockdowns had been implemented. It is possible that retrospective questions overstate the severity of the crisis on food security. Moreover, our measures also remain consistent in the survey modality through which they are measured (over the phone throughout), while many other studies use in-person measures from prior to the pandemic, and phone surveys thereafter, which might potentially bias responses in systematic ways.

The rural setting of our study might have also been protective. Rural areas were less likely to be affected by the virus, because of low population density, remoteness from population centers, and reliance on farming (which can be practiced at social distance), and so COVID itself did not spread widely in rural Africa. Perhaps some of these factors also insulated households from lockdowns—while economic activity in urban population centers clearly declined, this may not have spilled over into more rural areas. In our samples, people earn very little income to start with (less than $10 per month), and we find no evidence of a decline in income in Liberia. While we find a modest decline in Malawi, this appears to be mostly seasonal. Similarly, while markets were restricted, they were nevertheless open, and the price of food changed only modestly.

On the other hand, we also note that our study is not entirely unique in finding that food security levels did not dramatically change during the COVID-19 market restrictions. [21] analyze longitudinal data from five African countries, and find evidence supporting a worsening of food security in only one (Nigeria), leading them to conclude that a number of contextual factors, such as a terms of trade improvement in the case of Mali, and a bumper harvest in the case of Malawi, may have afforded protection from the pandemic, a finding that is mirrored in our work. [22] find that impacts of lockdowns were linked with market integration levels—farmer incomes declined in remote areas where market integration was poorer, but consumption improved because more stocks were available locally. Availability of food at the local level is also identified as a major determinant of pandemic-induced food insecurity by [3] in their review of more than 300 documents on the impacts of the lockdowns. Finally, we point to the evidence provided in [13] who show that while food security worsened nationally due to the pandemic in the country of Mali, only urban households identified the pandemic as the reason behind the lower levels of food security, suggesting that seasonal changes in food availability are an important consideration in rural areas, and that all changes in rural food security are not necessarily attributable to the pandemic.

Our findings are also similar to [23] and to [24], who find no worsening of food security in urban Addis Ababa and Delhi, respectively, despite a decrease in incomes; in the case of Delhi, however, this is driven by government-provided assistance. In our context, however, direct government support was non-existent: no households in our samples reported receiving any cash or food support from the government or an NGO during the lockdown. Also, unlike these papers, we do not observe a decline in income in our data, since our sample is made up largely of subsistence farmers. It is also worth noting that income declines will not necessarily lead to declines in food security as in the face of an income shock, households will likely reallocate expenditures from discretionary consumption towards essentials, such as food. For example, in the multi-country studies described in [11], even though there are declines in food security, they are, by and large, much smaller in magnitude than the declines in income. [25] also make this nuanced point by showing that in the wake of the pandemic, poor households in rural Kenya were able to preserve their level of food expenditure by reallocating spending away from schooling and transportation, as well as by cutting back on informal risk-sharing and delaying repayment of loans. Similarly, [26] show that while households in India had to reduce their consumption of vegetables and animal-source foods, they were able to largely maintain their consumption of staples, suggesting that the lockdowns largely impacted access to perishables. Therefore, the take-away from this set of papers should not be that poor households in developing countries have not been impacted by the pandemic, but simply that they have managed to preserve their *low* levels of food security during the pandemic, likely by cutting back on other expenditures.

Finally, we want to caveat our findings by noting that the results of this paper may be impacted by the fact that due to attrition, our phone survey sample is positively selected to be richer. As such, this is not a problem that is unique to our study as other papers that use panel surveys dating back to the pre-pandemic period face similar attrition issues [12, 13]. Nevertheless, our findings should be interpreted with caution.

## Conclusion

We document how households in rural Liberia and Malawi fared in the aftermath of the COVID-19 market disruptions using panel phone surveys of households and food vendors. We find high levels of awareness and behavior change and large declines in market activity. However, we find no evidence of increased food insecurity.

While our prior was that we would find large declines in food security, a plausible *ex post* reason for these results is that that rural areas like the ones we study are poorly connected to economic centers of activity; and while this may be a core reason for their poverty, paradoxically the isolation of these areas may make them more immune to economic declines in urban centers. We see evidence in favor of this both from the modest changes in prices, and in income (in large part because households earn so little income in the first place).

All in all, it appears that the worst fears about lockdowns were not realized, at least for these contexts. Our results suggest that lockdowns can be implemented in rural areas if necessary, without causing large increases in food insecurity (at least for some amount of time), even in very poor settings. The disease itself has not yet spread widely in much of rural Africa, and activities like subsistence farming have apparently continued with modest disruption. Other sources of income were disrupted for some time, but most households earned very little from such activities. Similarly, in other contexts, people have worried about the loss of services such as school meals—yet in this setting, kids were not getting meals in the first place anyway, so there was little to lose. (see S10 Table). In this context, market disruptions—which limit but do not eliminate economic activity, and not accompanied by a direct loss of assets—might be easier to cope with than natural disasters, even for very poor households.

## Supporting information

S1 Fig Timeline of project activities. (PDF)

S2 Fig Crop calendar of major food crops. This figure shows the cropping calendar for the major food crop in each country (maize in Malawi; rice in Liberia).
(PDF)

S3 Fig Historical price trends. This figure shows historical price trends for selected food items in Liberia and Malawi.
(PDF)

S4 Fig Timeline of government responses. (PDF)

S5 Fig Food security index component 1: Household dietary diversity score (z-score). This figure replicates our main result (Fig 2), for the first component of the food security index (the HDDS).
(PDF)

S6 Fig Food security index component 2: Food consumption score (z-score). This figure replicates our main result (Fig 2), for the second component of the food security index (the FCS).
(PDF)

S7 Fig Food security index component 3: Household hunger scale (z-score). This figure replicates our main result (Fig 2), for the third component of the food security index (the HHS).
(PDF)

S8 Fig Monthly household food expenditures in 2020 and 2021. This figure shows level changes across months and difference-in-differences coefficients for monthly household food expenditures in the year of the COVID lockdowns (2020) and the following year (2021).
(PDF)

S9 Fig Monthly household total expenditures in 2020 and 2021. This figure shows level changes across months and difference-in-differences coefficients for monthly household total expenditures in the year of the COVID lockdowns (2020) and the following year (2021).
(PDF)

S1 File Questionnaire on inclusivity in global research. (DOCX)

S1 Table Household phone survey attrition. This table shows attrition from the household phone surveys.
(PDF)

S2 Table Correlates of attrition from analysis sample. This table shows balance between those included in this paper’s analysis and those who are not.
(PDF)

S3 Table Correlates of attrition from balanced sample. This table shows the correlation between the proportion of bi-monthly surveys a household participated in and its baseline characteristics.
(PDF)

S4 Table Household characteristics in representative surveys. This table shows summary statistics from a representative sample of households in each country (the 2016 HIES in Liberia and the 2019 IHS5 in Malawi).
(PDF)

S5 Table Awareness and attitudes. This table shows descriptive information on awareness and attitudes regarding COVID-19.
(PDF)

S6 Table Change in business outcomes for food vendors. This table shows changes in selected business outcomes, for food vendors.
(PDF)

S7 Table Household food security index (z-score). This table shows regressions results for the household food security index (akin to the results shown in Fig 2).
(PDF)

S8 Table Crop prices. This table shows changes in crop prices. The odd-numbered columns utilize an event study design, and the even-numbered columns utilize a difference-in-differences (comparing 2020 to 2021).
(PDF)

S9 Table Non-agricultural income. This table shows regression results for non-agricultural income.
(PDF)

S10 Table School meals. This table shows descriptive information on school meals during the COVID lockdown period.
(PDF)

S1 Data (ZIP)
