# Supplementary figures and images for: COVID-19 market disruptions and food security: Evidence from households in rural Liberia and Malawi
Source: PLoS One. 2022 Aug 8;17(8):e0271488. doi: 10.1371/journal.pone.0271488 (PMC9359542; doi:10.1371/journal.pone.0271488)

# S1 Fig: Timeline of Project Activities

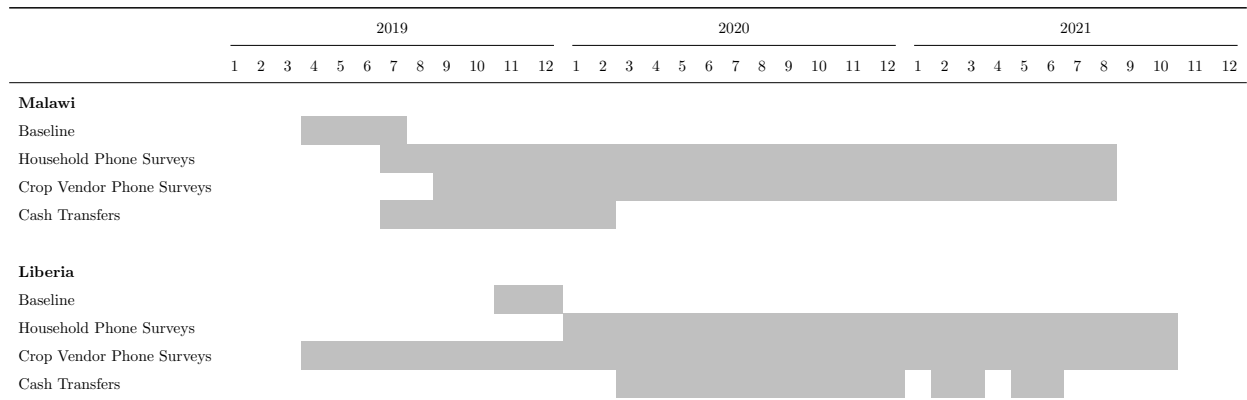

Supplement: S1 Fig — (PDF) [file pone.0271488.s001.pdf]

S4 Fig: Timeline of Government Responses

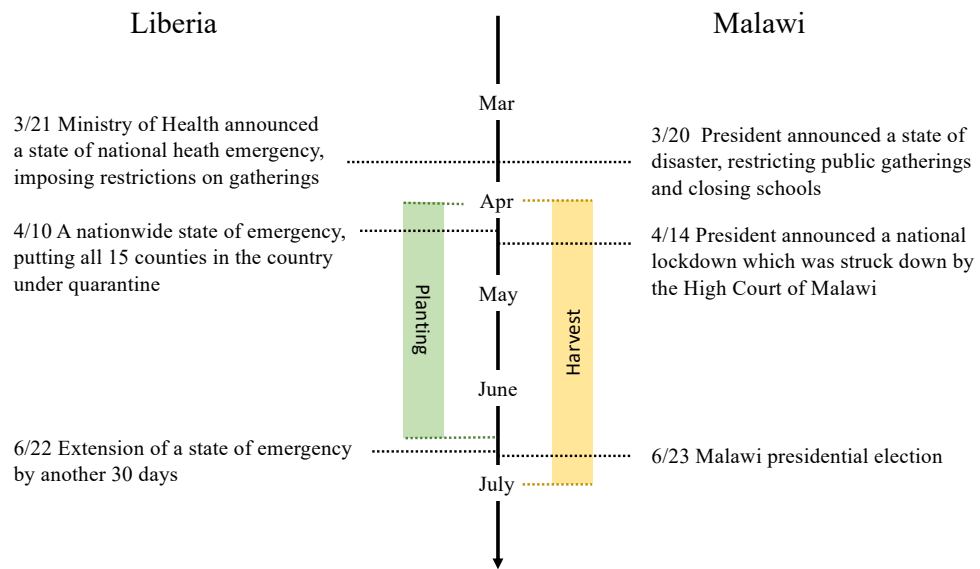

Supplement: S4 Fig — (PDF) [file pone.0271488.s004.pdf]
