# Supplementary material for: COVID-19 market disruptions and food security: Evidence from households in rural Liberia and Malawi
Source: PLoS One. 2022 Aug 8;17(8):e0271488. doi: 10.1371/journal.pone.0271488 (PMC9359542; doi:10.1371/journal.pone.0271488)
Supplement: S2 Fig — This figure shows the cropping calendar for the major food crop in each country (maize in Malawi; rice in Liberia). (PDF) [file pone.0271488.s002.pdf]

S2 Fig: Crop Calendar of Major Food Crops

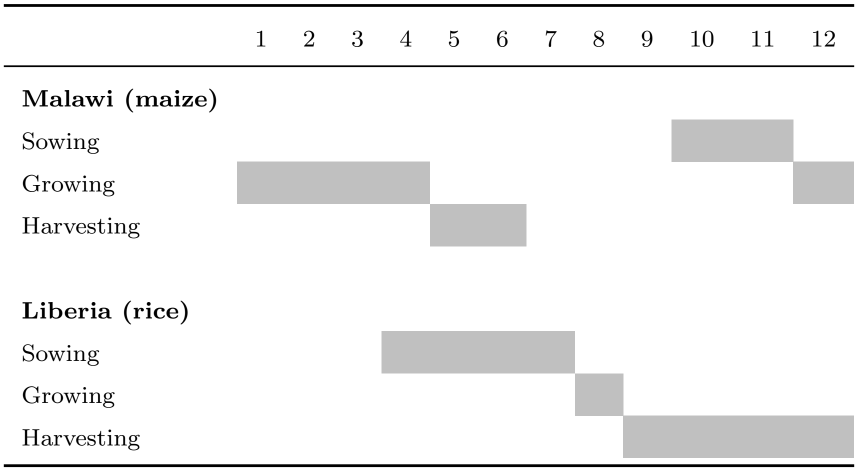

Source: FAO Global Information and Early Warning System (GIEWS). Lean season is November-February in Malawi and June-August in Liberia.
