# Supplementary material for: COVID-19 market disruptions and food security: Evidence from households in rural Liberia and Malawi
Source: PLoS One. 2022 Aug 8;17(8):e0271488. doi: 10.1371/journal.pone.0271488 (PMC9359542; doi:10.1371/journal.pone.0271488)
Supplement: S3 Fig — This figure shows historical price trends for selected food items in Liberia and Malawi. (PDF) [file pone.0271488.s003.pdf]

S3 Fig: Historical Price Trends

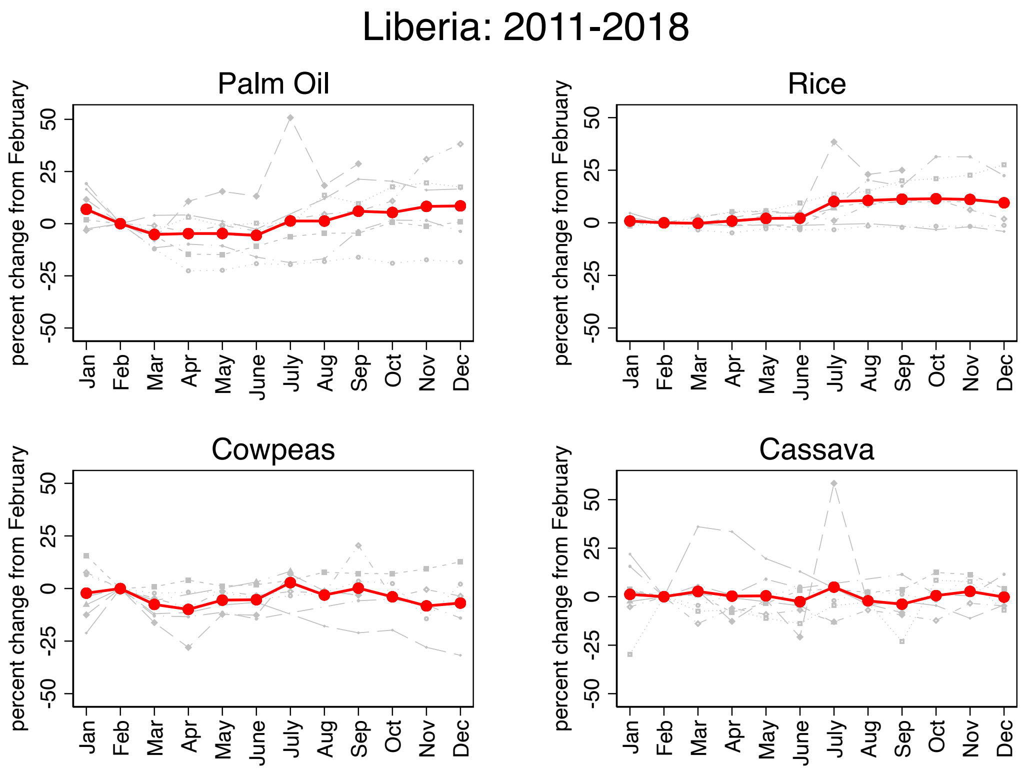

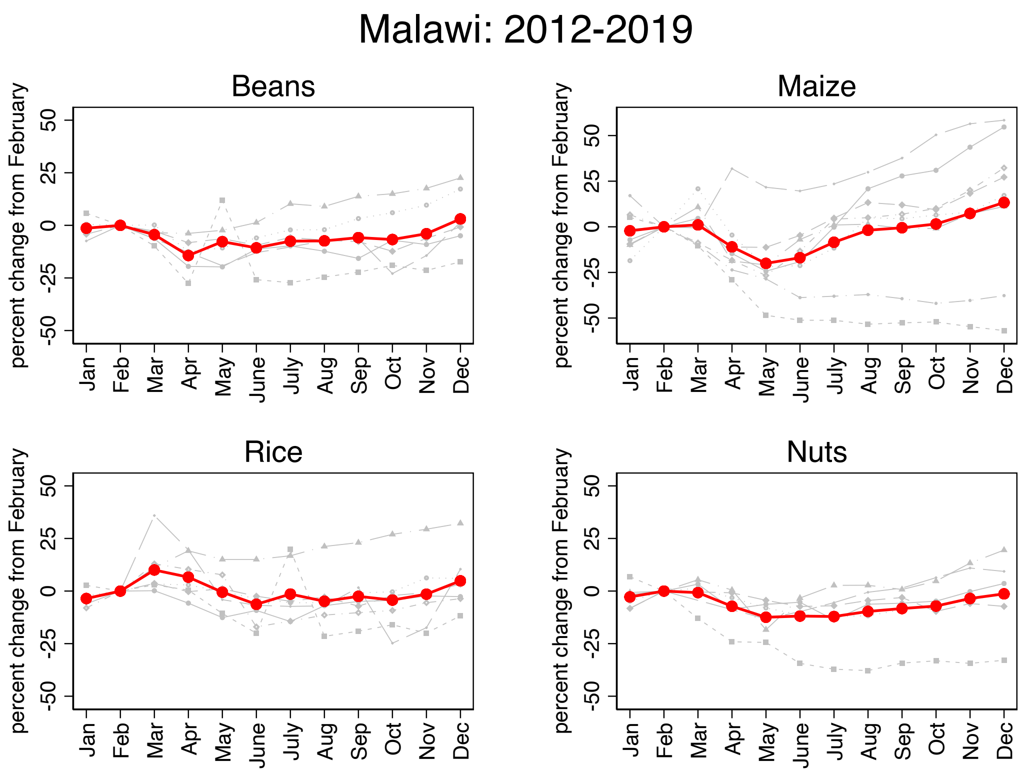

Note: Data is from the World Food Programme. For each month, we calculate the national average price, and calculate the percentage price change from February of the same year. Each grey line shows percent price change for an individual year, while the red present the average across years.
