# Supplementary material for: COVID-19 market disruptions and food security: Evidence from households in rural Liberia and Malawi
Source: PLoS One. 2022 Aug 8;17(8):e0271488. doi: 10.1371/journal.pone.0271488 (PMC9359542; doi:10.1371/journal.pone.0271488)
Supplement: S6 Fig — This figure replicates our main result (Fig 2), for the second component of the food security index (the FCS). (PDF) [file pone.0271488.s006.pdf]

S6 Fig: Food Security Index Component 2: Food Consumption Score (z-score)

Panel A: Liberia

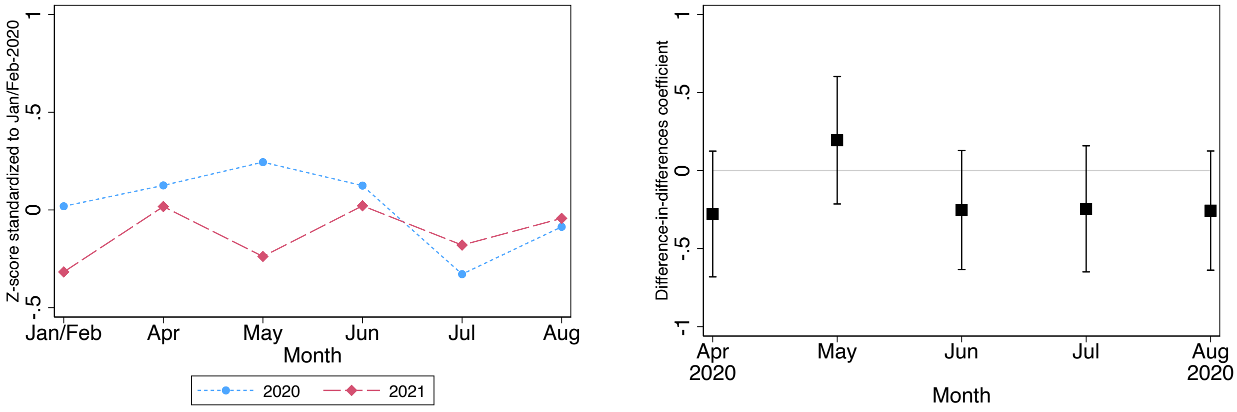

Panel B: Malawi

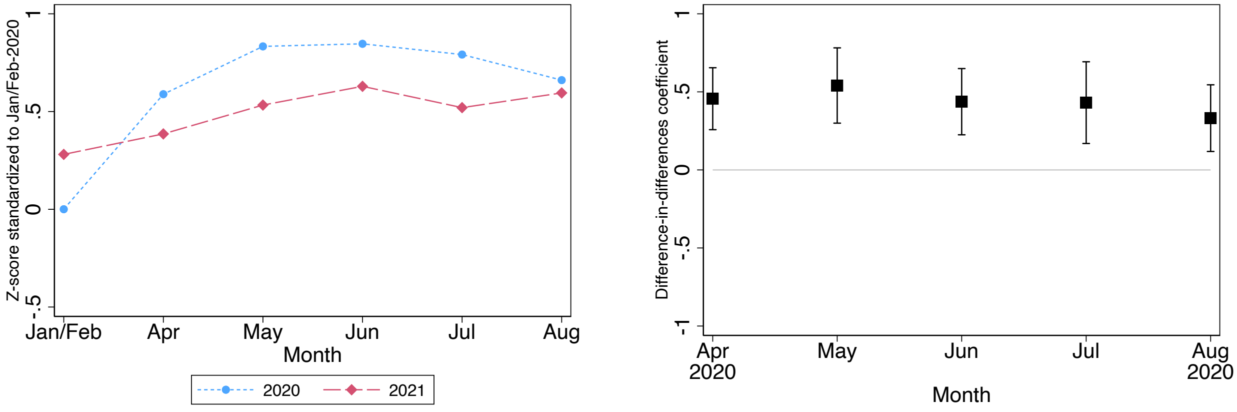

Note: This figure replicates our main result (Fig 2) for FCS, the second component of the Food Security Index.
