# Supplementary material for: COVID-19 market disruptions and food security: Evidence from households in rural Liberia and Malawi
Source: PLoS One. 2022 Aug 8;17(8):e0271488. doi: 10.1371/journal.pone.0271488 (PMC9359542; doi:10.1371/journal.pone.0271488)
Supplement: S7 Fig — This figure replicates our main result (Fig 2), for the third component of the food security index (the HHS). (PDF) [file pone.0271488.s007.pdf]

## S7 Fig: Food Security Index Component 3: Household Hunger Scale (z-score)

Panel A: Liberia

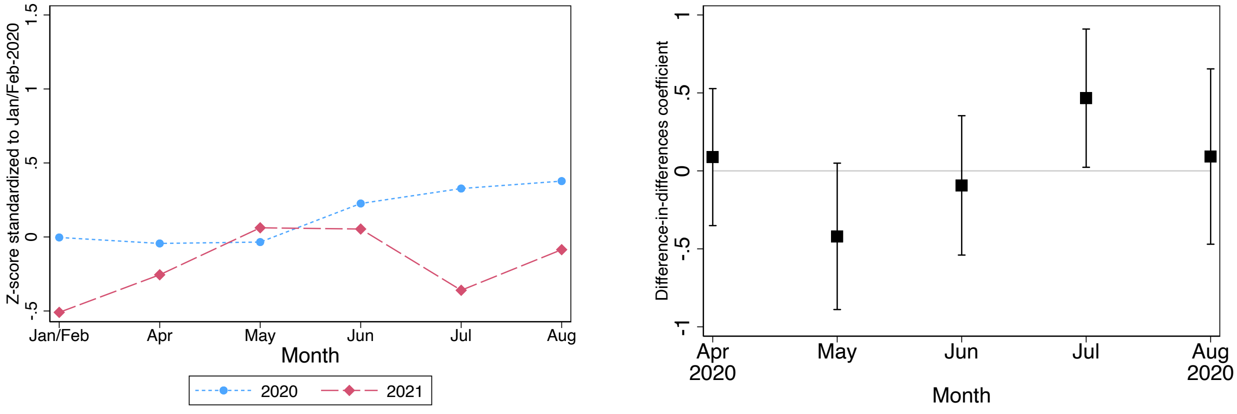


Panel B: Malawi

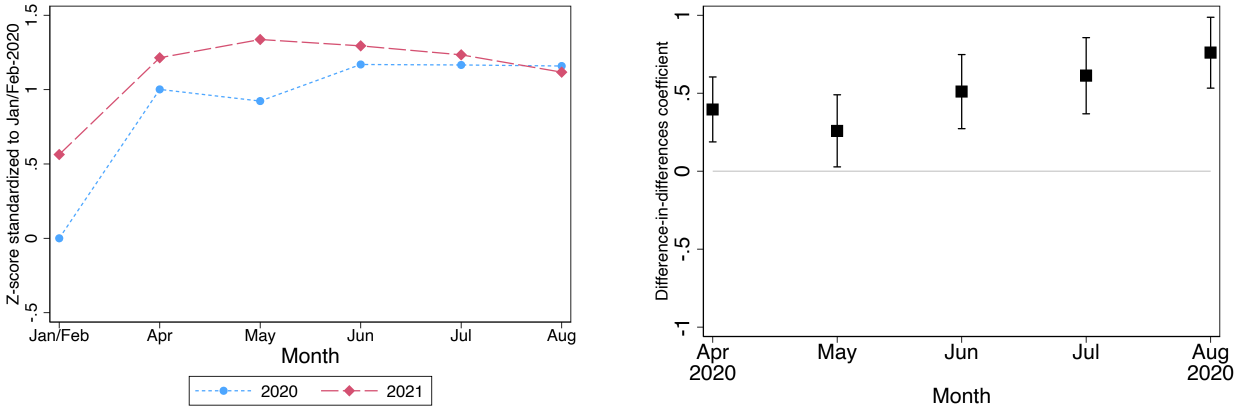


Note: This figure replicates our main result (Fig 2) for HHS, the third component of the Food Security Index.
