# Supplementary material for: COVID-19 market disruptions and food security: Evidence from households in rural Liberia and Malawi
Source: PLoS One. 2022 Aug 8;17(8):e0271488. doi: 10.1371/journal.pone.0271488 (PMC9359542; doi:10.1371/journal.pone.0271488)
Supplement: S9 Fig — This figure shows level changes across months and difference-in-differences coefficients for monthly household total expenditures in the year of the COVID lockdowns (2020) and the following year (2021). (PDF) [file pone.0271488.s009.pdf]

S9 Fig: Monthly Household Total Expenditures in 2020 and 2021

Panel A: Liberia

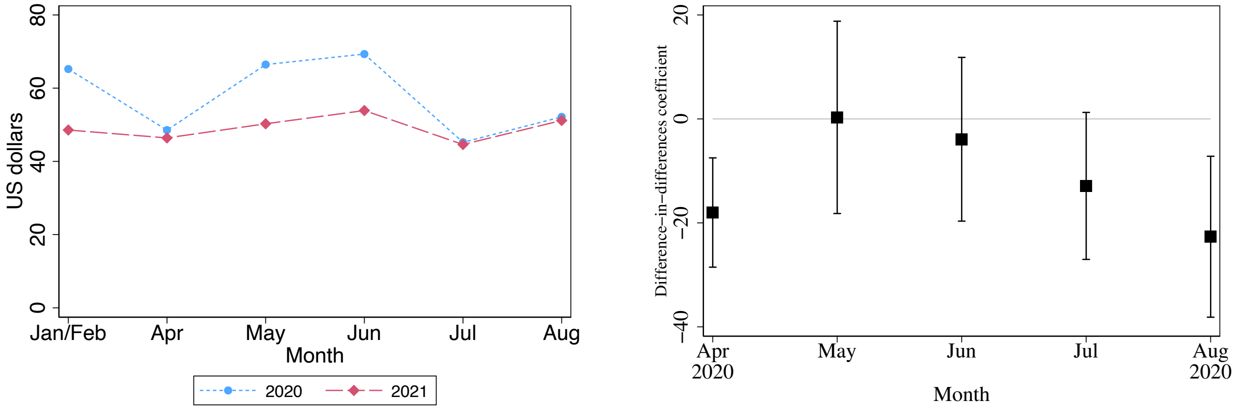

Panel B: Malawi

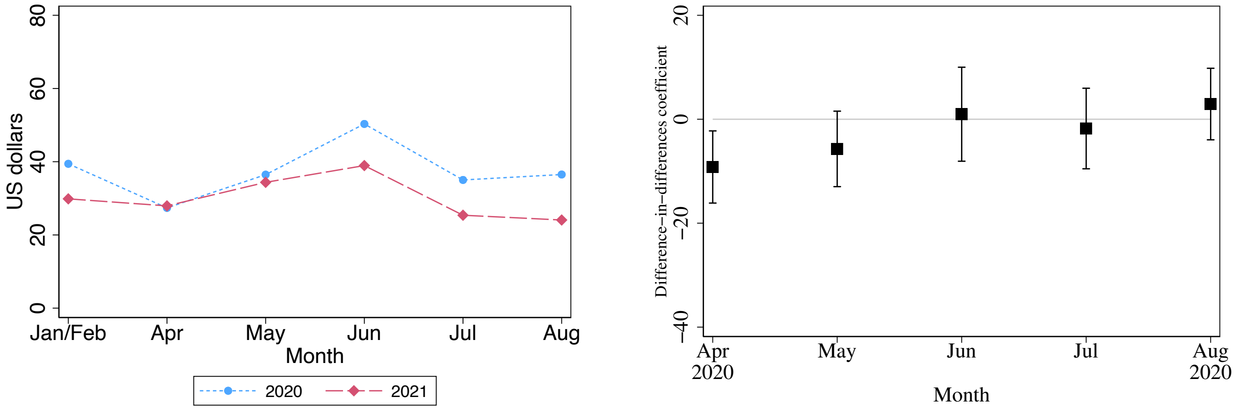

Note: Outcome is monthly total expenditure (including food expenditure) in USD. Subfigures on the left plot the change in levels across months, while those on the right report coefficients (with 95% confidence intervals) from the difference-in-differences specification in Eq 3. Regressions include household-by-calendar-month fixed effects. Standard errors are clustered at the village level.
