# Supplementary material for: COVID-19 market disruptions and food security: Evidence from households in rural Liberia and Malawi
Source: PLoS One. 2022 Aug 8;17(8):e0271488. doi: 10.1371/journal.pone.0271488 (PMC9359542; doi:10.1371/journal.pone.0271488)
Supplement: S1 Table — This table shows attrition from the household phone surveys. (PDF) [file pone.0271488.s011.pdf]

S1 Table: Household Phone Survey Attrition

	(1)	(2)	(3)	(4)	(5)	(6)	(7)	(8)	(9)	(10)	(11)	(12)
	=1 if completed survey in following month:											
	Jan	Feb	Mar	Apr	May	Jun	Jul	Aug	Sep	Oct	Nov	Dec
**Panel A: Liberia**												
2020	0.67	0.82	0.77	0.61	0.70	0.70	0.56	0.60	0.61	0.69	0.63	0.59
2021	0.60	0.53	0.50	0.54	0.49	0.50	0.50	0.46				
**Panel B: Malawi**												
2020	0.89	0.90	0.91	0.92	0.91	0.97	0.97	0.94	0.95	0.97	1.00	0.77
2021	0.79	0.89	0.87	0.83	0.83	0.81	0.81	0.77				

Note: The Liberia results (Panel A) include only Wave 2 of the study (since coverage over this time period was spotty in Wave 1). There are 206 respondents in Liberia and 297 respondents in Malawi. Half of the phone survey sample was called for each month.
