# Supplementary material for: COVID-19 market disruptions and food security: Evidence from households in rural Liberia and Malawi
Source: PLoS One. 2022 Aug 8;17(8):e0271488. doi: 10.1371/journal.pone.0271488 (PMC9359542; doi:10.1371/journal.pone.0271488)
Supplement: S2 Table — This table shows balance between those included in this paper’s analysis and those who are not. (PDF) [file pone.0271488.s012.pdf]

S2 Table: Correlates of Attrition from Analysis Sample

	(1)	(2)
	Dep. variable: =1 if in analysis sample	
	Liberia	Malawi
**Panel A: Demographics**		
=1 if female	-0.06	-0.04***
	(0.07)	(0.01)
Age (divided by 10)	0.04*	0.00
	(0.02)	(0.01)
=1 if currently married or has partner	-0.05	0.02
	(0.08)	(0.03)
Years of education (divided by 10)	0.02	0.04
	(0.08)	(0.03)
Number of household members (divided by 10)	-0.13	0.02
	(0.14)	(0.05)
**Panel B: Expenditure and assets**		
Household monthly expenditure (Thou. USD)	0.33	0.19*
	(0.60)	(0.10)
Household food expenditure (Thou. USD)	0.92	0.76
	(1.84)	(0.51)
=1 if respondent has access to mobile phone	0.25***	0.01
	(0.05)	(0.02)
=1 if house owned	-0.08	0.03
	(0.06)	(0.04)
=1 if house has thatch roof	-0.20**	-0.05**
	(0.09)	(0.02)
Total value of land and housing (Thou. USD)	-0.05	0.01***
	(0.08)	(0.00)
Total value of physical assets (Thou. USD)	0.33	0.11
	(1.13)	(0.10)
Net value of financial assets (Thou. USD)	1.41	-0.49
	(1.44)	(0.43)
**Panel C: Food security**		
For any household member in the past month:		
=1 if skipped a meal	0.11*	-0.02
	(0.06)	(0.02)
=1 if went to sleep hungry	0.12*	-0.03
	(0.06)	(0.02)
=1 if had no food for an entire day	0.08	-0.01
	(0.08)	(0.03)
Mean Dependent variable	0.73	0.96
Observations	206	297

Note: The table shows coefficients from bivariate regressions for each covariate using compliance as dependent variable. Compliance is defined as 1 if the household ever appears in the analysis of this paper, and 0 otherwise. Standard errors are clustered at the village level.
