# Supplementary material for: COVID-19 market disruptions and food security: Evidence from households in rural Liberia and Malawi
Source: PLoS One. 2022 Aug 8;17(8):e0271488. doi: 10.1371/journal.pone.0271488 (PMC9359542; doi:10.1371/journal.pone.0271488)
Supplement: S3 Table — This table shows the correlation between the proportion of bi-monthly surveys a household participated in and its baseline characteristics. (PDF) [file pone.0271488.s013.pdf]

S3 Table: Correlates of Attrition from Balanced Sample

	(1)	(2)
	Dep. variable: % of survey rounds completed	
	Liberia	Malawi
**Panel A: Demographics**		
=1 if female	-0.07**	-0.04
	(0.03)	(0.03)
Age (divided by 10)	0.01	0.00
	(0.01)	(0.01)
=1 if currently married or has partner	-0.02	0.01
	(0.03)	(0.02)
Years of education (divided by 10)	0.01	0.02
	(0.04)	(0.03)
Number of household members (divided by 10)	0.02	0.03
	(0.06)	(0.04)
**Panel B: Expenditure and assets**		
Household monthly expenditure (Thou. USD)	0.64**	0.26
	(0.27)	(0.16)
Household food expenditure (Thou. USD)	0.11	1.32***
	(0.82)	(0.47)
=1 if respondent has access to mobile phone	0.09***	0.02
	(0.03)	(0.02)
=1 if house owned	0.05	0.05**
	(0.03)	(0.02)
=1 if house has thatch roof	-0.04	-0.01
	(0.05)	(0.02)
Total value of land and housing (Thou. USD)	0.03	0.01
	(0.05)	(0.00)
Total value of physical assets (Thou. USD)	0.22	0.18***
	(0.45)	(0.06)
Net value of financial assets (Thou. USD)	-0.57	-0.53
	(0.70)	(0.55)
**Panel C: Food security**		
For any household member in the past month:		
=1 if skipped a meal	0.00	-0.01
	(0.03)	(0.02)
=1 if went to sleep hungry	0.02	-0.02
	(0.03)	(0.02)
=1 if had no food for an entire day	0.01	-0.03
	(0.04)	(0.02)
Mean Dependent variable	0.75	0.85
Observations	150	285

Note: The sample is restricted to households which appear in the analysis of this paper. Each entry is a separate bivariate regression, in which the dependent variable is the proportion of survey rounds in which the household completed a phone survey. Standard errors are clustered at the village level.
