# Supplementary material for: COVID-19 market disruptions and food security: Evidence from households in rural Liberia and Malawi
Source: PLoS One. 2022 Aug 8;17(8):e0271488. doi: 10.1371/journal.pone.0271488 (PMC9359542; doi:10.1371/journal.pone.0271488)
Supplement: S4 Table — This table shows summary statistics from a representative sample of households in each country (the 2016 HIES in Liberia and the 2019 IHS5 in Malawi). (PDF) [file pone.0271488.s014.pdf]

S4 Table: Household Characteristics in Representative Surveys

	(1)	(2)	(3)	(4)	(5)	(6)	(7)	(8)
	Liberia HIES 2016				Malawi IHS5 2019-2020			
	Rural		Study Area		Rural		Study Area	
	Mean	SD	Mean	SD	Mean	SD	Mean	SD
**Panel A: Demographics**								
Age	43.92	35.89	42.96	13.36	43.86	16.75	43.14	17.18
=1 if currently married or has partner	0.82		0.81		0.69		0.64	
Years of education	2.42	2.35	2.39	2.21	5.57	2.52	5.27	2.39
Number of household members	5.37	2.19	5.51	2.22	4.43	2.04	4.28	1.95
**Panel B: Income, expenditure, and assets**								
=1 planted any crop	0.89		0.99		0.40		0.79	
=1 if sold any harvest	0.56		0.52		0.30		0.44	
=1 if owns a business enterprise	0.21		0.25		0.57		0.19	
=1 if household owns a mobile phone	0.35		0.46		0.70		0.50	
=1 if house owned	0.66		0.85		0.52		0.75	
=1 if house has thatch roof	0.26		0.51		0.12		0.47	
Household food expenditure	20.22	17.23	14.94	15.68	23.87	17.18	10.15	10.18
Business income (USD)	3.27	13.54	2.31	10.69	89.19	368.63	21.09	72.44
Total value of physical assets	74.26	137.59	98.26	190.71	518.99	1,060.31	275.62	808.58
Durable goods (USD)	26.22	65.27	54.28	126.10	490.56	1,055.90	196.58	709.54
Livestock (USD)	48.04	113.69	43.98	116.95	28.43	143.87	79.04	298.43
**Panel C: Food security**								
=1 if insufficient food in past 12 months	0.92		0.88		0.53		0.68	
Household dietary diversity score (0-12)	5.76	1.88	5.51	1.67	8.93	1.80	8.44	2.22
Food Consumption Score (0-112)	48.17	15.86	46.22	15.07	54.07	16.18	44.30	17.12
Observations	5570		740		9342		688	

Note: This table presents summary statistics from representative, living standards household surveys (the 2016 Liberia HIES and the 2019-2020 Malawi IHS5). In columns 1-2 and 5-6, statistics are restricted to rural households. In Liberia, the study areas are Bong and Nimba counties; in Malawi, they are Chiradzulu and Machinga districts. Sample weights are used to recover population estimates. All monetary values are in USD and winsorized at the 99th percentile.
