# Supplementary material for: COVID-19 market disruptions and food security: Evidence from households in rural Liberia and Malawi
Source: PLoS One. 2022 Aug 8;17(8):e0271488. doi: 10.1371/journal.pone.0271488 (PMC9359542; doi:10.1371/journal.pone.0271488)
Supplement: S5 Table — This table shows descriptive information on awareness and attitudes regarding COVID-19. (PDF) [file pone.0271488.s015.pdf]

S5 Table: Awareness and Attitudes

	(1) Liberia	(2) Malawi
**Panel A: Basic awareness**		
=1 if respondent:		
is aware of recent virus outbreak	0.98	0.99
knows that it's called coronavirus/COVID-19	0.90	1.00
thinks it's a real public health problem	0.95	0.98
**Panel B: Opinions about government response to COVID**		
Do you trust the information central gov't is providing?[a]	4.37	4.71
Do you trust the information local leaders are providing?[a]	4.39	4.68
=1 if central gov't and local leaders give different info	0.05	0.12
Do you think gov't measures to prevent spread are helpful?[b]	4.24	4.10
Do you approve gov't measures?[c]	4.19	4.09
**Panel C: Support from government/NGO during lockdown**		
=1 if received cash transfers		0.00
=1 if received food support		0.00
**Panel D: Concern about COVID contraction**		
=1 if worried or extremely worried about COVID contraction	0.87	0.93
=1 if knows anyone who tested for COVID-19	0.04	0.09

Note:
[a] 0-5 scale index. 0 - have not received any guidelines; 1 - don't trust at all; 2 - somewhat distrust; 3 - neither trust nor distrust; 4 - somewhat trust; 5 - trust completely.
[b] 1-5 scale index. 1 - not at all helpful; 2 - not helpful; 3 - neither helpful nor not helpful; 4 - helpful; 5 - very helpful.
[c] 1-5 scale index. 1 - strongly disapprove; 2 - disapprove; 3 - neither approve nor disapprove; 4 - approve; 5 - strongly approve.
