# Supplementary material for: COVID-19 market disruptions and food security: Evidence from households in rural Liberia and Malawi
Source: PLoS One. 2022 Aug 8;17(8):e0271488. doi: 10.1371/journal.pone.0271488 (PMC9359542; doi:10.1371/journal.pone.0271488)
Supplement: S6 Table — This table shows changes in selected business outcomes, for food vendors. (PDF) [file pone.0271488.s016.pdf]

S6 Table: Change in Business Outcomes for Food Vendors

	(1) No. of customers (daily)	(2) Revenue (monthly)	(3) Profit (monthly)	(4) Other income (monthly)
**Panel A: Liberia**				
April	-14.41***	-101.60***	-24.63***	-4.62**
	(0.79)	(11.61)	(2.43)	(2.18)
May	-12.20***	-101.52***	-18.91***	-2.82
	(0.92)	(20.69)	(3.84)	(2.40)
June	-10.34***	-86.52***	-14.13***	-1.33
	(1.09)	(23.79)	(4.45)	(2.43)
February mean	31.66	256.58	47.53	7.73
February SD	21.39	405.33	69.56	55.20
F statistic	114.76	30.43	40.45	3.22
Observations	2,200	2,200	2,200	2,200
No. of vendors	676	676	676	676
**Panel B: Malawi**				
April	-22.12***	-182.01***	-44.34***	-17.50***
	(1.64)	(30.12)	(4.92)	(6.23)
May	-23.80***	-136.60***	-32.20***	2.64
	(2.44)	(50.38)	(6.82)	(8.46)
June	-24.88***	-118.67**	-21.73***	3.40
	(2.40)	(48.05)	(7.68)	(6.86)
February mean	67.68	592.57	103.59	34.80
February SD	73.82	1276.22	196.02	188.23
F statistic	61.53	12.73	28.49	5.95
Observations	3,813	3,813	3,813	3,813
No. of vendors	1,042	1,042	1,042	1,042

Note: All variables are winsorized at the 99th percentile. All monetary values are in USD. Standard errors are clustered at the market level.
