# Supplementary material for: COVID-19 market disruptions and food security: Evidence from households in rural Liberia and Malawi
Source: PLoS One. 2022 Aug 8;17(8):e0271488. doi: 10.1371/journal.pone.0271488 (PMC9359542; doi:10.1371/journal.pone.0271488)
Supplement: S7 Table — This table shows regressions results for the household food security index (akin to the results shown in Fig 2). (PDF) [file pone.0271488.s017.pdf]

S7 Table: Household Food Security Index (z-score)

	(1)	(2)	(3)	(4)	(5)	(6)
	Liberia			Malawi		
	Event Study	Difference-in Difference		Event Study	Difference-in Difference	
April 2020	0.18	-0.20	-0.23	1.12***	0.52***	0.52***
	(0.13)	(0.17)	(0.19)	(0.09)	(0.10)	(0.10)
May 2020	0.10	-0.03	-0.01	1.01***	0.51***	0.51***
	(0.14)	(0.21)	(0.24)	(0.09)	(0.11)	(0.11)
June 2020	0.39***	-0.15	-0.24	1.40***	0.63***	0.61***
	(0.14)	(0.16)	(0.18)	(0.09)	(0.11)	(0.11)
July 2020	-0.03	0.16	0.23	1.22***	0.72***	0.73***
	(0.15)	(0.18)	(0.21)	(0.09)	(0.11)	(0.11)
Aug 2020	0.39**	0.17	0.27	1.38***	0.75***	0.81***
	(0.16)	(0.18)	(0.21)	(0.09)	(0.10)	(0.10)
Jan-Feb 2020 mean	0.00	0.00	-0.01	0.00	0.00	0.02
Jan-Feb 2020 SD	1.00	1.00	1.03	1.00	1.00	1.00
Observations	456	808	634	960	1,795	1,635
No. of households	150	150	129	285	285	272

Note: This table shows regression results for Fig 2. The dependent variable is the Food Security Index (z-score). Columns 1 and 4 report coefficients from the event study regressions as specified in Eq 1 using the data for 2020. Columns 2 and 5 show difference-in-difference regressions specified in Eq 2; columns 3 and 6 display results from Eq 3 (i.e., the right-hand-side subfigures in Fig 2). All regressions include household fixed effects and standard errors clustered at the village level. Columns 3 and 6 additionally include household-by-calendar-month fixed effects, so that we compare the same set of households for each calendar month in 2020 and 2021.
