# Supplementary material for: COVID-19 market disruptions and food security: Evidence from households in rural Liberia and Malawi
Source: PLoS One. 2022 Aug 8;17(8):e0271488. doi: 10.1371/journal.pone.0271488 (PMC9359542; doi:10.1371/journal.pone.0271488)
Supplement: S8 Table — This table shows changes in crop prices. The odd-numbered columns utilize an event study design, and the even-numbered columns utilize a difference-in-differences (comparing 2020 to 2021). (PDF) [file pone.0271488.s018.pdf]

S8 Table: Crop Prices

	(1)	(2)	(3)	(4)	(5)	(6)	(7)
	All Items		Staples		Rice/Maize		
	Dep. Variable: Ratio of Price to Price in February of Same Year						
**Panel A: Liberia**							
April 2020	0.338***	0.339***	0.170***	0.194***	0.070***	0.088***	0.061***
	(0.042)	(0.045)	(0.022)	(0.028)	(0.011)	(0.016)	(0.011)
May 2020	0.377***	0.320***	0.201***	0.076**	0.072***	-0.040**	0.051***
	(0.044)	(0.058)	(0.025)	(0.033)	(0.012)	(0.020)	(0.012)
June 2020	0.329***	0.317***	0.171***	0.185***	0.049***	0.083***	0.027**
	(0.041)	(0.052)	(0.026)	(0.033)	(0.012)	(0.016)	(0.012)
July 2020	0.312***	0.244***	0.177***	0.181***	0.107***	0.119***	0.005
	(0.044)	(0.052)	(0.025)	(0.029)	(0.014)	(0.019)	(0.014)
August 2020	0.304***	0.229***	0.151***	0.164***	0.095***	0.108***	-0.011
	(0.037)	(0.043)	(0.021)	(0.026)	(0.013)	(0.019)	(0.013)
2020		0.000		0.003		0.002	
		(0.035)		(0.024)		(0.013)	
Controls for Price Trend	N	N	N	N	N	N	Y
Markets	80	80	80	80	80	80	80
Observations	479	951	1,354	2,756	473	931	473
Feb 2020 Mean (USD)	0.871	0.871	0.539	0.539	0.681	0.681	0.681
**Panel B: Malawi**							
April 2020	-0.226***	-0.156***	-0.254***	-0.147***	-0.535***	-0.458***	-0.425***
	(0.019)	(0.022)	(0.012)	(0.018)	(0.013)	(0.019)	(0.013)
May 2020	-0.197***	-0.131***	-0.263***	-0.056***	-0.518***	-0.198***	-0.318***
	(0.015)	(0.020)	(0.013)	(0.017)	(0.013)	(0.016)	(0.013)
June 2020	-0.203***	-0.129***	-0.239***	-0.031*	-0.512***	-0.220***	-0.342***
	(0.015)	(0.018)	(0.012)	(0.018)	(0.010)	(0.019)	(0.010)
July 2020	-0.201***	-0.131***	-0.231***	-0.034*	-0.493***	-0.204***	-0.409***
	(0.013)	(0.019)	(0.012)	(0.017)	(0.010)	(0.018)	(0.010)
August 2020	-0.167***	-0.133***	-0.183***	-0.038**	-0.469***	-0.265***	-0.450***
	(0.014)	(0.017)	(0.012)	(0.017)	(0.012)	(0.020)	(0.012)
2020		-0.001		-0.001		-0.001	
		(0.013)		(0.012)		(0.012)	
Controls for Price Trend	N	N	N	N	N	N	Y
Markets	95	95	95	95	95	95	95
Observations	567	1,135	2,179	4,312	566	1,117	566
Feb 2020 Mean (USD)	1.111	1.111	0.680	0.680	0.489	0.489	0.489

Note: The dependent variable is the ratio of the price in the given month to the price in February of the same year. Columns 1-2 show a price index, where units are weighted based on the expenditure share in the baseline survey. Columns 3-4 show a similar index for staples only. Columns 5-7 show the price of the main staple (rice in Liberia and maize in Malawi). The unit of observation is the market-month. Data includes prices from January-August 2020 and January-August 2021 (not including March in either year). All prices are in USD and winsorized at the 1st and 99th percentiles. Odd columns show event study regressions including only 2020 prices, and even columns difference-in-differences regressions including both 2020 and 2021 prices. Standard errors are clustered at the market level. Regressions include product fixed effects and market fixed effects. Staple crops in Liberia are local rice, imported rice, cassava, and cassava flour. Staple crops in Malawi are sweet potatoes, maize, maize flour, beans, and pigeon peas. Columns 5-7 refer to imported rice for Liberia (Panel A) and maize for Malawi (Panel B). Additionally, in Column 7, the long run monthly average prices for February from the WFP 2011-2019 price database are used to calculate the price ratio.
