# Supplementary material for: COVID-19 market disruptions and food security: Evidence from households in rural Liberia and Malawi
Source: PLoS One. 2022 Aug 8;17(8):e0271488. doi: 10.1371/journal.pone.0271488 (PMC9359542; doi:10.1371/journal.pone.0271488)
Supplement: S9 Table — This table shows regression results for non-agricultural income. (PDF) [file pone.0271488.s019.pdf]

S9 Table: Non-agricultural Income

	(1)	(2)	(3)	(4)	(5)	(6)
	Business profit		Casual Labor income		Other Non-agricultural income	
**Panel A: Liberia**						
April 2020	1.01	0.45	3.98***	1.64	3.67***	2.45
	(0.93)	(1.39)	(1.29)	(1.77)	(1.25)	(1.74)
May 2020	-2.72***	-2.25	1.79	4.35*	1.77	4.81*
	(0.96)	(1.38)	(1.69)	(2.61)	(1.62)	(2.62)
June 2020	0.03	-1.21	0.96**	-1.24	0.82**	-0.19
	(0.67)	(1.05)	(0.45)	(1.07)	(0.38)	(0.95)
July 2020	-2.82***	-2.50**	1.57	3.42*	1.94	3.28*
	(0.87)	(0.96)	(1.45)	(2.00)	(1.34)	(1.92)
August 2020	-0.55	-1.43	1.70*	-2.49	1.07*	-2.43
	(0.91)	(1.10)	(0.86)	(2.41)	(0.64)	(2.21)
Jan-Feb 2020 mean	3.34	3.25	2.62	2.50	1.38	1.37
Jan-Feb 2020 SD	7.27	7.37	6.67	6.10	6.21	5.66
Observations	456	634	456	634	456	634
No. of households	150	129	150	129	150	129
**Panel B: Malawi**						
April 2020	0.23	-0.36	-3.78***	-1.55	-0.01	0.34
	(0.40)	(0.56)	(0.76)	(1.05)	(0.03)	(0.42)
May 2020	-0.11	-0.70	-2.82***	0.04	-0.27	0.12
	(0.37)	(0.65)	(0.81)	(0.93)	(0.30)	(0.46)
June 2020	0.22	-0.51	-2.73***	-0.92	0.02	-0.35
	(0.36)	(0.61)	(0.81)	(1.17)	(0.09)	(0.48)
July 2020	0.25	-1.00	-2.64***	0.74	-0.14	0.01
	(0.38)	(0.61)	(0.74)	(1.07)	(0.32)	(0.50)
August 2020	0.69*	-0.26	-3.07***	-0.40	0.39*	0.04
	(0.41)	(0.56)	(0.78)	(1.01)	(0.21)	(0.56)
Jan-Feb 2020 mean	0.94	0.99	6.55	6.55	0.26	0.28
Jan-Feb 2020 SD	3.75	3.87	8.48	8.40	2.21	2.32
Observations	960	1,635	960	1,635	960	1,635
No. of households	285	272	285	272	285	272

Note: The dependent variable is monthly income in USD. Odd columns show event study regressions, and even columns difference-in-differences regressions. All regressions include household fixed effects. Standard errors clustered at the village level. Difference-in-difference regressions additionally include household-by-calendar-month fixed effects. All monetary variables are in USD and winsorized at the 99th percentile.
