# Supplementary material for: COVID-19 market disruptions and food security: Evidence from households in rural Liberia and Malawi
Source: PLoS One. 2022 Aug 8;17(8):e0271488. doi: 10.1371/journal.pone.0271488 (PMC9359542; doi:10.1371/journal.pone.0271488)
Supplement: S10 Table — This table shows descriptive information on school meals during the COVID lockdown period. (PDF) [file pone.0271488.s020.pdf]

S10 Table: School Meals

	(1) Liberia	(2) Malawi
=1 if following meals were provided in school (before closure):		
breakfast	0.03	0.56
lunch	0.33	0.01
snack	0.01	0.00
no food at all	0.52	0.37
=1 if respondent reported yes to:		
children miss out meals	0.23	0.12
respondent spends money to make more food	0.56	0.76
assitance from family/neighbor/friends	0.01	0.00
assistance from village chief/gov't/aid programs	0.33	0.13

Note: Questions were asked of all households and crop vendors with school-aged children. N=2,029 (507 in Liberia and 1,522 in Malawi).
