# Supplementary material for: COVID-19 market disruptions and food security: Evidence from households in rural Liberia and Malawi
Source: PLoS One. 2022 Aug 8;17(8):e0271488. doi: 10.1371/journal.pone.0271488 (PMC9359542; doi:10.1371/journal.pone.0271488)
Supplement: S1 Data — (ZIP) [file pone.0271488.s021.zip › readme.docx]

To replicate and generate all figures and tables in the manuscript and in the appendix, run < replication_dofile.do>. The do file uses following input data sets, which we describe below in detail.

1. **Primary data sets collected by authors in the field**

Note that not all data sets have unique data because some data sets have been processed to make them a particular shape that is convenient to run analysis.

baseline_analysis_fullsample.dta

- Level: household
- Main variables: baseline food security and household expenditure

cvps_price_analysis.dta

- Level: a monthly panel of crop vendor phone surveys
- Main variables: crop prices

fig3.dta

- Level: a monthly panel of crop vendor phone surveys
- Main variables: Crop prices

figures.dta

- Level: a monthly panel of household phone surveys
- Main variables: food security and household expenditure

hhps_analysis_studysample.dta

- Level: a monthly panel of household phone surveys for the analysis sample
- Main variables: food security and household expenditure

hhps_analysis_fullsample.dta

- Level: a monthly panel of household phone surveys for full phone survey sample
- Main variables: income and household expenditure

covid_vars_cleaned.dta

- Level: a pooled version of (i) monthly panel of crop vendor surveys and (ii) monthly panel of household phone surveys
- Main variables: covid awareness and attitudes, crop vendor business outcomes, and school meals

fig1.dta

- Level: a pooled version of (i) household baseline and (ii) household phone surveys
- Main variables: food security and household expenditure

table1.dta

- Level: household
- Main variables: baseline household demographics, expenditure and assets, and food security

table2.dta

- Level: a pooled version of (i) monthly panel of crop vendor surveys and (ii) monthly panel of household phone surveys
- Main variables: economic activities, behavior changes, and business disruptions on crop vendors

1. **Secondary data sets collected from public sources**

LSMS2019_HIES2016_LR_MW.dta

- Source: World Bank’s Living Standard Measurements Survey for Malawi (2019) and Household Income and Expenditure Survey for Liberia (2016)
- Level: household
- Main variables: household demographics, Income, expenditure and assets, and food security

Appendix_fig_S3.dta

- Source: World Food Program
- Level: country-market-crop-year-month
- Main variables: crop prices

These replication files were verified to work under STATA 16.1
